# Supplementary material for: Lives of Skin Lesions in Monkeypox: Histomorphological, Immunohistochemical, and Clinical Correlations in a Small Case Series
Source: Viruses. 2023 Aug 15;15(8):1748. doi: 10.3390/v15081748 (PMC10458687; doi:10.3390/v15081748)
Supplement: Supplementary file 1 [file viruses-15-01748-s001.zip › viruses-2541180-Figure S1-updated-8.15.pdf]

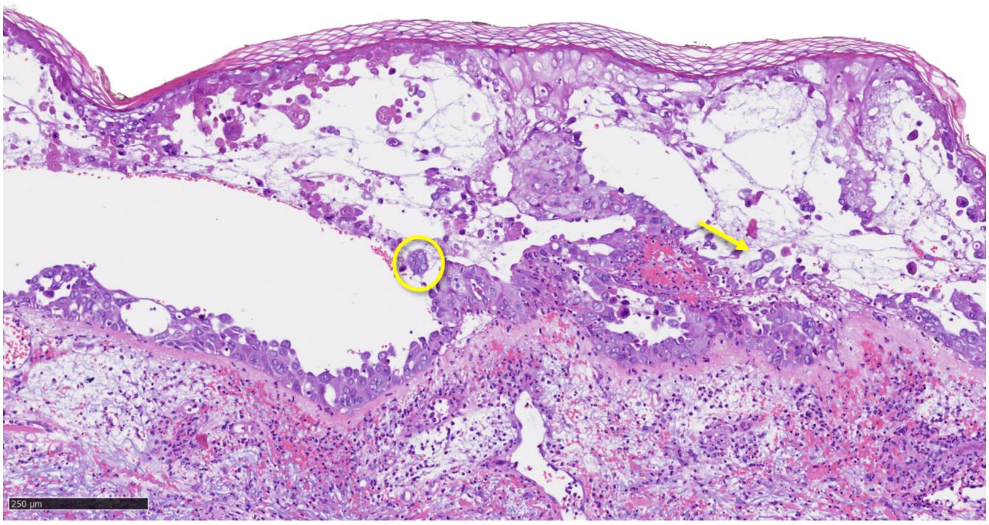

Supplementary Figure S1. Histological picture of a herpes infection of the skin. Extensive acantholysis with multinucleated cells (yellow circle) and evidence of steel-gray nuclei with marginalization of chromatin (yellow arrow) support a diagnosis of herpes infection. Stain: hematoxylin and eosin, scale bar 250 µm
